# Analysis of cellular autofluorescence in touch samples by flow cytometry: implications for front end separation of trace mixture evidence

**DOI:** 10.1007/s00216-017-0364-0

**Published:** 2017-05-18

**Authors:** M. Katherine Philpott, Cristina E. Stanciu, Ye Jin Kwon, Eduardo E. Bustamante, Susan A. Greenspoon, Christopher J. Ehrhardt

**Affiliations:** 1 Department of Forensic Science, Virginia Commonwealth University, 1015 Floyd Avenue, Richmond, VA 23284 USA; 2 Virginia Department of Forensic Science, 700 North 5th Street, Richmond, VA 23219 USA

**Keywords:** Biological mixtures, Flow cytometry, Touch, Trace, FACS, Epithelial cells, Mixture interpretation, STR profiling, Autofluorescence

## Abstract

The goal of this study was to survey optical and biochemical variation in cell populations deposited onto a surface through touch or contact and identify specific features that may be used to distinguish and then sort cell populations from separate contributors in a trace biological mixture. Although we were not able to detect meaningful biochemical variation in touch samples deposited by different contributors through preliminary antibody surveys, we did observe distinct differences in red autofluorescence emissions (650–670 nm), with as much as a tenfold difference in mean fluorescence intensities observed between certain pairs of donors. Results indicate that the level of red autofluorescence in touch samples can be influenced by a donor’s contact with specific material prior to handling the substrate from which cells were collected. In particular, we observed increased red autofluorescence in cells deposited subsequent to handling laboratory gloves, plant material, and certain types of marker ink, which could be easily visualized microscopically or using flow cytometry, and persisted after hand washing. To test whether these observed optical differences could potentially be used as the basis for a cell separation workflow, a controlled two-person touch mixture was separated into two fractions via fluorescence-activated cell sorting (FACS) using gating criteria based on intensity of 650–670 nm emissions and then subjected to DNA analysis. Genetic analysis of the sorted fractions provided partial DNA profiles that were consistent with separation of individual contributors from the mixture suggesting that variation in autofluorescence signatures, even if driven by extrinsic factors, may nonetheless be a useful means of isolating contributors to some touch mixtures.

**Electronic supplementary material:**

The online version of this article (doi:10.1007/s00216-017-0364-0) contains supplementary material, which is available to authorized users.

**Graphical Abstract:**
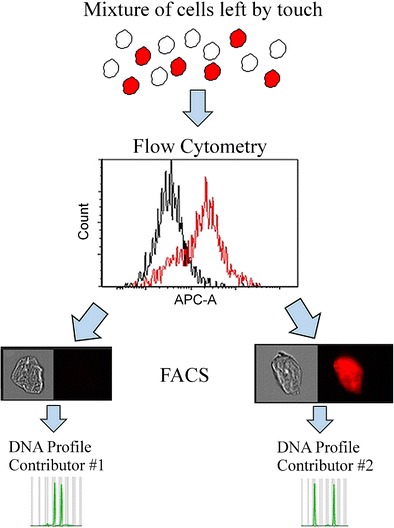
Conceptual workflow diagram. Trace biological mixtures containing cells from multiple individuals are analyzed by flow cytometry. Cells are then physically separated into two populations based on intensity of red autofluorescence using Fluorescence Activated Cell Sorting. Each isolated cell fraction is subjected to DNA analysis resulting in a DNA profile for each contributor.

## Introduction

Analysis of “touch” or trace epithelial cell mixtures poses a significant challenge for forensic DNA caseworking units. Currently, interpretation of mixed short tandem repeat (STR) profiles developed from touched surfaces requires time-consuming and frequently subjective procedures that not only decrease the probative value of the evidence (sometimes completely) but also have been the recent subject of heavy criticism for lacking scientific validity [1]. Although probabilistic genotyping systems can perform analyses on complex mixtures which are superior to human analysis, implementation of these systems poses a number of challenges (e.g., cost; time and resource requirements for validation; legal skirmishes over proprietary software; difficulties associated with communicating probabilistic information to a jury), mis-estimation of the number of contributors to a sample can affect probabilistic results [2], and there are limits as to the number of contributors that can be successfully disentangled [3]. There remains a considerable need for front end techniques that can separate cell populations from different contributors prior to DNA analysis thereby facilitating the generation of single source STR profiles and/or simplifying multi-contributor samples [4].

Toward this end, a number of methods exist for isolating cells from a mixture including laser capture microdissection, microfluidic manipulation, and fluorescence-activated cell sorting [5–8]. These techniques typically take advantage of either morphological or immunochemical variation between cells and have proven to be effective for resolving uncompromised mixtures with multiple cell types (e.g., blood-saliva [8], sperm-epithelial [7, 9]) as well as mixtures of multiple individuals contributing a single cell type [5]. However, there has been limited published research on front end separation strategies on “touch” mixture samples consisting solely or largely of epidermal cells which have vastly different biological and structural properties from other forensically relevant cell types (e.g., vaginal, buccal, white blood cells).

Specifically, sloughed epidermal cells (aka corneocytes) come from the outermost layer of the skin, the stratum corneum. Prior to shedding, corneocytes have undergone a process of terminal differentiation as they migrate to the outer layer whereby they have lost their cellular organelles including their nuclei [10, 11]. Additionally, various keratin molecules accumulate both within and upon the surface of the cell as it becomes encased within an extracellular matrix composed of lipids and hydrophobic proteins that contribute to the skin’s barrier function [12, 13]. The progressive keratinization of epidermal cells and degradation of their intracellular components may pose considerable obstacles to the development of immunochemical techniques to differentiate contributors in touch mixture samples. Surface antigens, which can be targeted for selective labeling of individual cell populations in some types of forensic mixture [5, 8, 9], have variable reactivity in keratinocytes originating from different epidermal layers (e.g., basal layer is more reactive than spinous layer) [14–16], and their utility has yet to be explicitly evaluated in touch samples.

It is well established that some biological molecules exhibit native fluorescence that can be visualized without the need for immunohistochemical techniques, including components of skin such as tryptophan, porphyrins, and melanins. Non-biological compounds comprised of aromatic rings or multiple pi bonds may also fluoresce. In the clinical context, such autofluorescence is often viewed as an obstacle to methodologies involving detection of specific fluorescent signals; however, in certain cases—including some dermatological conditions—autofluorescence can be a useful diagnostic tool [17, 18]. The extent to which fluorescence signatures—whether endogenous or exogenous, biological or non-biological—may allow for differentiation in touch samples remains unexplored.

Therefore, the objective of this study was to characterize the immunochemistry and optical properties of cells recovered from touched surfaces with the overarching goal of identifying biomolecular targets or optical signatures that may be used to differentiate, and ultimately separate, epidermal cell populations from different individuals. We initially focused on the reactivity of epidermal cells to antibody probes that target two different protein classes: the human leukocyte antigen (HLA) complex and cytokeratins (CKs). We followed these experiments with a survey of autofluorescence of epidermal cells from different contributors at red wavelengths (650–670 nm). This wavelength was chosen based on preliminary surveys of unstained touch cell samples [19] where inter-contributor differences were observed. We then tested the effect that handling different substances prior to touch sample deposition had on the fluorescence profile of contributors’ cells.

Next, we used observed inter-contributor variation in autofluorescence profiles to develop gating criteria for subsequent fluorescence-activated cell sorting (FACS) in an effort to physically isolate contributor cell populations in a controlled two-person “touch” mixture. Finally, we processed the sorted cell populations using standard forensic DNA analysis methods and compared the STR profiles of each fraction against profiles from the unsorted mixture and the contributor reference samples in order to evaluate the efficacy of separation.

## Methods

### Collection of touch samples for antibody hybridization, imaging flow cytometry, and conventional flow cytometry

Touch samples were obtained pursuant to VCU-IRB approved protocol ID no. HM20000454_CR3. Volunteers were asked to rub a sterile polypropylene conical tube (P/N 229421; Celltreat Scientific) using their palm and fingers for 5 min. Cells were collected from the surface with sterile pre-wetted swabs (P/N 22037924; Fisher Scientific) followed by dry swabs. A total of six wet swabs and two dry swabs were used to sample the entire tube surface. To elute the cells into solution, the swabs were manually stirred then vortexed for 15 s in 10 mL of ultrapure water (18.2 MΩ cm). The entire solution was then passed through a 100-μm filter mesh prior to antibody hybridization, conventional flow cytometry, and imaging flow cytometry (IFC). Separate aliquots of the resulting cell solution were used for each analysis method.

### Collection of touch samples after handling specific materials

For studies of exogenous influences on autofluorescence in touch samples, each donor handled a specific material prior to depositing cells on a conical tube. These materials included purple nitrile gloves (Precision® brand, powder-free, P/N PCS775), plant material, and conical tubes marked with Sharpie® marker ink. Prior to handling these materials, donors washed their hands with antibacterial soap under running water for 15 s and then allowed them to air dry. For nitrile glove experiments, donors wore a nitrile glove on their right hand, leaving the left (control) hand bare, and proceeded to grip/handle various items with their gloved hand for 5 min to simulate normal activity (e.g., pipette, door handle, tools). The glove was then removed and the contributor held a conical tube in each hand for 5 min. For experiments involving plant material, subjects handled individual leaves of kale or collard greens using only their right hand for 5 min (left hand was not used and served as a control cell population). The handling procedure involved lifting/tossing leaves with fingers and palmar surface and tearing individual leaves into smaller pieces, approximating how this material might be handled during food preparation. Subsequently, donors rinsed their hands with water for approximately 5 s (to remove pieces of plant material) and allowed their hands to air dry before depositing touch samples by holding a conical tube in each hand for 5 min. For marker ink experiments, each donor held a conical tube that had been marked with a black or green marker in his/her right hand for 5 min before depositing touch samples by holding an unmarked conical tube in each hand for 5 min. For each of these experiments, cells were collected from the surface of each tube and eluted into solution as described above. Separate aliquots of the resultant cell solutions were used for flow cytometry analysis and IFC.

### Collection of touch samples after handling material and hand washing

To test the effect that hand washing has on exogenous sources of autofluorescence in contributor cell populations, donors were asked to manipulate kale leaves with both hands (lifting, tossing, and tearing, as described above). Each donor then held a wooden-handled kitchen knife with one hand for 5 min. Next, the donors washed both hands with soap and water for 15–20 s and allowed their hands to air dry. Finally, each donor held a second knife in his or her other hand (i.e., the hand that was not used to hold the first knife) for 5 min. Cells were collected from the handle of each kitchen knife as described above. Cell solutions derived from the unwashed hand were compared to cell solutions from the hand that had been washed immediately prior to deposition using flow cytometry as well as IFC. Because both hands were used to handle plant material during this experiment, negative control cell populations from the same donor (where cells were deposited without any immediate prior contact with plant material) were collected on a separate day and analyzed using flow cytometry.

### Collection of touch samples for mixture studies

To test whether observed variations in autofluorescence between two touch sample contributors could be used to successfully separate a mixture of their cells, each donor rubbed a conical tube as described above. The surface of each donor’s tube was swabbed with one slightly wetted cotton-tipped swab followed by one dry swab. The swabs were then eluted in 2 mL of sterile water, vortexed for 15 s, and passed through a 100-μm mesh filter. An 860-μL aliquot of each donor’s touch cell solution was combined to create a 1:1 mixture (by vol.) for flow cytometry analysis, gating, and subsequent sorting via FACS. Another 200 μL from each donor was combined to create a mixture that would proceed directly to DNA analysis without sorting (i.e., to develop an unsorted mixture profile for comparison). The remaining cell solution for each of the two donors was utilized for IFC studies.

### Antibody hybridization

Three-milliliter aliquots of donors’ touch cell solutions were centrifuged at 5,000×*g* for 5 min. The resulting cell pellets were then dissolved in ~100 μL of supernatant and incubated for 10 min with 1 μL of human Fc receptor block (Cat. no. 130-059-901, Miltenyi Biotec) to increase the specificity of antibody binding before reaction with either HLA or CK probes. For HLA hybridizations, cells were incubated with mouse anti-human monoclonal antibody (mAb) HLA-ABC-FITC (Cat. no. 311403, BioLegend) for 30 min. Cells incubated with anti-mouse IgG2a-FITC (Cat. no. 343303, BioLegend) for 30 min served as the isotype control for these experiments. Cells were then washed once in 1× FACS buffer [PBS supplemented with 2% fetal bovine serum (FBS, Cat. no. 100-106, Gemini BioProducts) and 10% sodium azide (Cat. no. S2002, Sigma-Aldrich)] and resuspended in the same solution until flow cytometry analysis.

For CK hybridization experiments, cells were incubated with anti-acidic cytokeratin probe (“AE1” (recognizes CKs 10, 14, 15, 16 and 19), Cat. no. 14-9001-80, Affymetrix eBioscience) for 30 min followed by reaction with a secondary antibody, anti-mouse IgG1-APC (Cat. no. 17-4015-80, Affymetrix eBioscience). We used anti-mouse IgG1-APC (Cat. no. 17-4714-42, Affymetrix eBioscience) to create the isotype control for AE1 experiments, incubating for 30 min. As before, cells were washed once and then resuspended in 1× FACS buffer prior to analysis.

### Imaging flow cytometry

For fluorescence imaging, intact epidermal cells were first isolated from ~500-μL aliquots of touch sample cell solutions by sorting the “large cell” fraction (i.e., “K” subpopulation in forward scatter (FSC)–side scatter (SSC) plots described in [20] into a collection tube using a BD FACSAria™ II (Becton Dickinson) flow cytometer with 488- and 633-nm coherent solid state lasers and set to the following channel voltages: FSC, 200 V; SSC, 475 V. The sorted cell solution (containing at least 1000 events) was then analyzed using an Amnis® Imagestream X Mark II (EMD Millipore) equipped with 488- and 642-nm lasers. Images of individual events were captured in the brightfield channel and allophycocyanin (APC) channel (642–745 nm). Magnification and focus settings varied with cell size. Cell images were analyzed and exported with the IDEAS® Software (EMD Millipore).

### Flow cytometry and fluorescence-activated cell sorting

For HLA and CK studies, flow cytometry analysis was performed on the BD FACSCanto™ II Analyzer (Becton Dickinson) equipped with 488- and 633-nm lasers. Channel voltages were set as follows: FSC, 150 V; SSC, 200 V; Alexa Fluor 488 (FITC), 335 V; phycoerythrin (PE), 233 V; PE-Cy5, 300 V; PE-Cy7, 400 V; and APC, 250 V. For each experiment, 10,000 total events were collected for analysis. Data analysis was performed using FCS Express 4 Flow Research Edition (De Novo Software).

Intrinsic fluorescence studies of touch samples and FACS of two-person epidermal cell mixtures were performed on one of two BD FACSAria™ II (Becton Dickinson) flow cytometers, each employing 488- and 633-nm coherent solid-state lasers. On each instrument, channel voltages were set as follows: FSC, 200 V; SSC, 475 V; and APC, 400 V. Events falling into the “large cell” gate were analyzed for red autofluorescence (650–670 nm), again using FCS Express 4 Flow Research Edition. Comparisons between fluorescence intensity histograms were generally made for the distribution of events fluorescing between 1 and 10^4^ relative fluorescent units (RFUs). For mixture samples, sorting gates were set to enrich for each of the two contributors in the mixture based on their individual autofluorescence profiles (“P9” and “P10” regions of the fluorescence histograms shown in Fig. 6). The majority of the cell solution aliquot (1720 μL) was processed through FACS, with a small amount left unprocessed to prevent introduction of air bubbles.

### DNA extraction, purification and quantitation

Sorted samples were centrifuged at 10,000×*g* for 15–20 min to pellet cells. The supernatant was concentrated onto a YM-100 Microcon filter (P/N 42413, EMD Millipore) and eluted in 25 μL of sterile distilled water, then re-combined with the cell pellet. These samples as well as the unsorted mixture sample and reference samples (donor buccal samples) were each lysed and purified using the DNA IQ System (Cat. no. DC6701, Promega) following the Virginia Department of Forensic Science (VA-DFS) standard protocols (Virginia Department of Forensic Science 2015). DNA extracts were quantitated using the Plexor HY System kit (Cat. no. DC1001, Promega) coupled with the Stratagene MX3005P Quantitative PCR Instrument and Plexor Analysis Software.

### STR amplification and profiling

We used the PowerPlex® Fusion System kit (Cat. no. DC2402, Promega) to amplify STRs in an ABI 9700 thermal cycler, following the manufacturer’s protocols. Capillary electrophoresis was performed on the ABI 3500 xL Genetic Analyzer (Life Technologies) as described in the instruction manual, and resulting data was analyzed using GeneMapper ID® X v1.4 Software (Life Technologies) according to the manufacturer’s recommendations. The analytical thresholds used to interpret the resulting data were dye-specific and set at 88 RFUs for fluorescein, 74 for JOE, 114 for the TMR-ET, and 80 for CXR-ET. The stochastic threshold was set at 396 RFU.

## Results and discussion

### Antibody labeling experiments

Cells hybridized to pan-HLA probe (recognizing all antigens within the A, B, and C protein classes) displayed no increase in average fluorescence when compared to unlabeled cells or isotype controls (see Electronic Supplementary Material (ESM); compare Fig. S1c to Fig. S1a, b). Similar results were obtained when HLA probes specific for the A*02 allele were hybridized against cells that screened positive for the A*02 allele (data not shown). These results suggest that HLA antigens were either not present or were unreactive on touch epidermal cells. The absence of HLA probe interactions observed in this study is consistent with these touch samples being comprised primarily of fully differentiated keratinocytes, which have been shown to display limited reactivity to HLA class I probes in contrast to cells derived from deeper layers of the epidermis [15, 16] or non-epidermal epithelial cell sources [21].

Experiments using cytokeratin probe AE1 showed probe uptake for each donor sample tested when compared against unstained cells and isotype controls (see ESM; compare Fig. S1f to Fig. S1d, e). We observed slight inter-individual variation in binding efficiency, but all donors exhibited overlap in their histogram profiles. These preliminary results suggest that cytokeratin expression—at least on the pan-level that is capable of being explored with a probe such as AE1—may not present a useful means of discriminating between epidermal cells from different donors. Future research efforts might benefit from focusing on individual cytokeratins, which may be differentially expressed in skin based on factors such as age [22].

### Intrinsic fluorescence surveys

Following these initial results with probe-based systems, we turned to cellular autofluorescence as a potentially discriminating characteristic for touch cell populations from different individuals. Specifically, we focused on red wavelengths (~650–670 nm (APC channel)) based on initial observations in the course of antibody hybridization studies that unstained cell samples from some contributors showed higher mean fluorescence intensities at these wavelengths than others (see ESM, Fig. S1d, e (maroon histograms), and data provided in [19]).

**Fig. 1:**
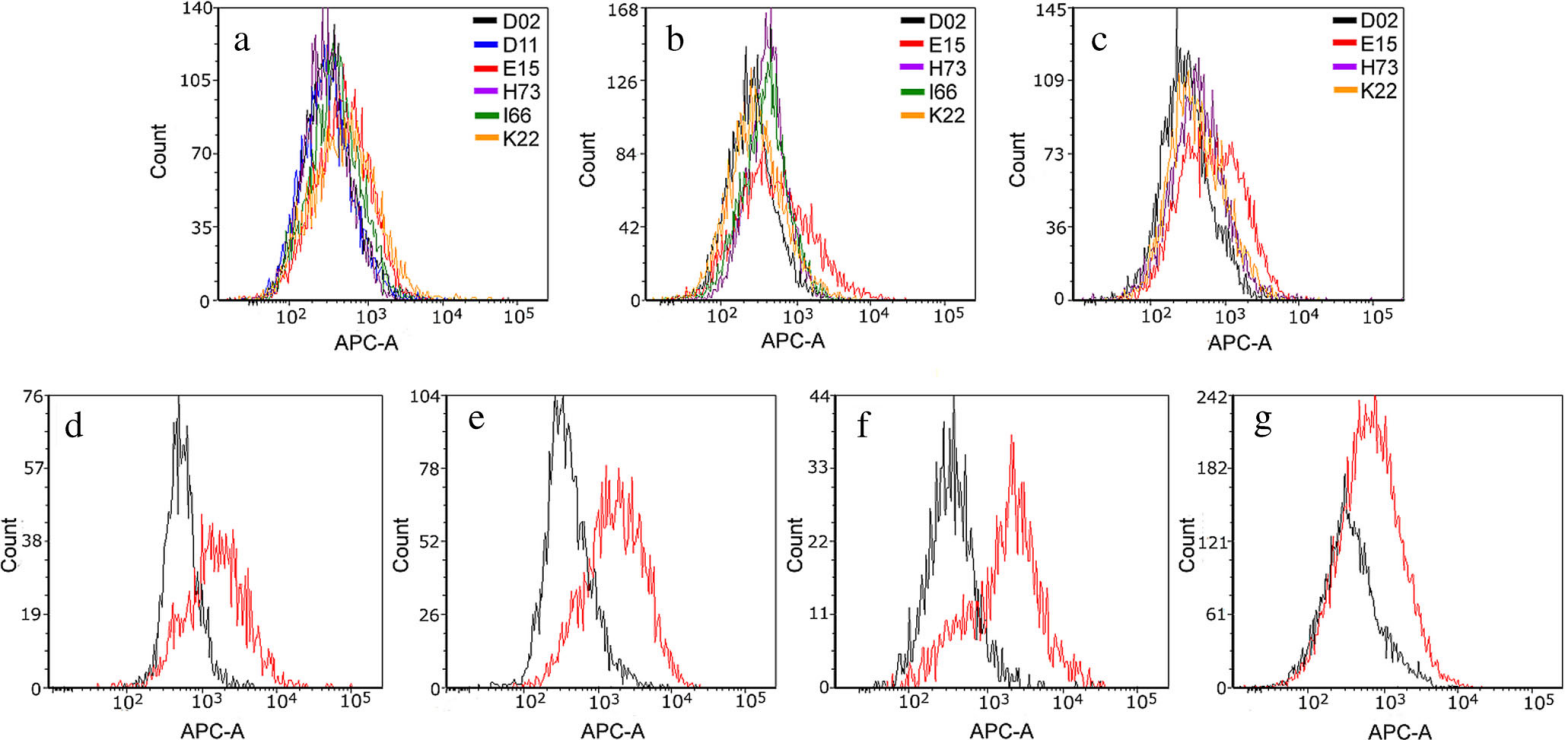
Overlaid red fluorescence (650–670 nm) histograms for cell populations from touch samples. Each panel (**a**–**c**) shows a different combination of donor cell populations sampled and analyzed on the same day. Panels **d**–**g** show overlaid red fluorescence histograms for two contributors, D02 (*black*) and E15 (*red*), across four additional sampling days. For these and all other histograms presented in this paper, the *X*-axis is fluorescence (in RFUs) and the *Y*-axis is cell count

Accordingly, we monitored the APC channel autofluorescence from donors’ touch samples, with subsets of these individuals sampled and analyzed on three different days; results are shown overlaid and grouped by sampling day in Fig. 1a–c. Overlap was observed between many of the donors on each sampling day. However, touch samples from one contributor, E15 (red histogram in panels a–c), consistently contained a number of flow events with higher fluorescence intensity than those from other contributors. Microscopic surveys of touch samples from donors D02 and E15 showed red autofluorescence of varying intensity largely associated with what appear to be intact corneocytes (see ESM, Fig. S2, compare cell images collected from donor E15 (e.g., frames 13, 20 (bright), 24, 34, 40, 46, 47 (lower intensity but still observable)) with images from donor D02 (dim fluorescence generally associated with fragments, not intact cells)).

To investigate the consistency of autofluorescence signatures, touch samples were collected from donors E15 and D02 on four additional days and analyzed for red autofluorescence. The degree of differentiation (or conversely overlap) between autofluorescence profiles varied across days, although the APC channel fluorescence values of E15 cell populations were consistently higher than D02 populations (Fig. 1d–g). Respective cell yield of donors appeared to work in concert with fluorescence differences to influence the degree of overlap observed: where the number of flow events captured from the “low-fluorescence” contributor (D02) was small relative to the “high-fluorescence” contributor (E15), there was complete overlap of D02’s contribution (compare Fig. 1g to Fig. 1d–f). Note that the distribution of fluorescence intensities is consistently broader for the “high”-fluorescence contributor, with a variable number of cells fluorescing at low intensities comparable to the “low-fluorescence” contributor, and other cells fluorescing more brightly.

The observation that red autofluorescence varied between donors led us to explore the possible causes for this phenomenon. Due to differences observed in the histograms developed from touch samples collected from the same donor on different days, we hypothesized that contact with exogenous substances prior to depositing a touch sample might play a role in the observed variation. We investigated this by executing a series of controlled experiments where donors handled specific materials encountered in the laboratory (nitrile gloves, marker ink) or at home (plant material) immediately prior to depositing a touch sample.

**Fig. 2:**
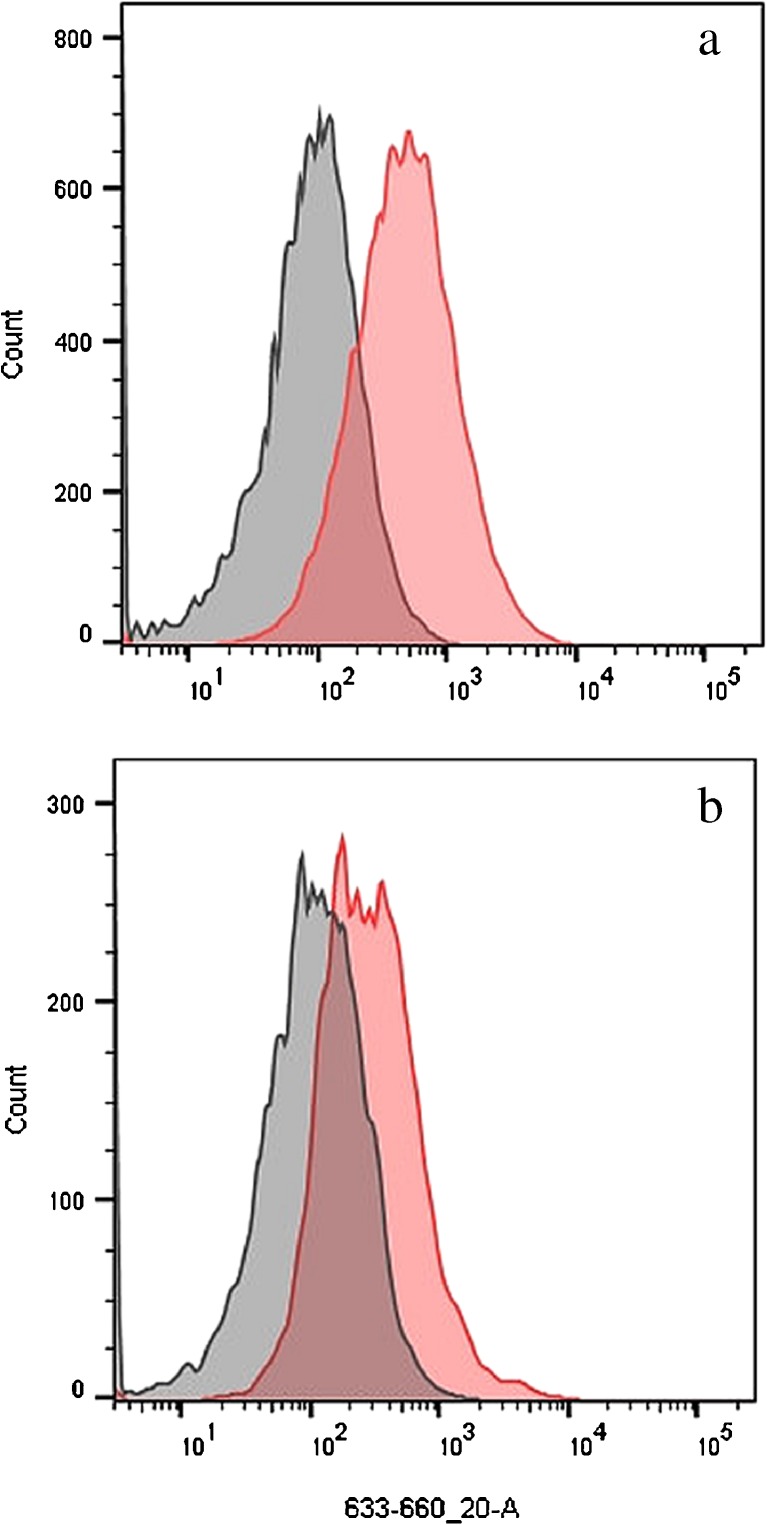
Overlaid APC channel histograms for samples generated from donors handling substrates bearing marker ink prior to cell deposition. Panels **a** and **b** show cell populations from the right and left hand of the same contributor sampled on two different days that handled a substrate previously written on with marker. Only one hand (*red histogram*) handled marked substrate leaving the other hand as a control (*gray histogram*)

We observed shifts in red autofluorescence of touch cell samples subsequent to handling each of the tested materials, with the degree of shift depending on the material handled. Histograms of touch samples collected from a donor after handling an item bearing marker ink on two different days (black ink 1 day, green ink the other) displayed shifted red fluorescence intensity histograms compared to cell populations from the control (i.e., non-marker) hand (Fig. 2). While overlap was observed in the fluorescence intensities of a subset of cells from the marker and control touch samples, fluorescence-based sorting gates can be conceived that should capture a significant number of events from one cell population to the exclusion or near exclusion of the other (e.g., one fraction <100 RFU, second fraction >1000 RFU).

**Fig. 3:**
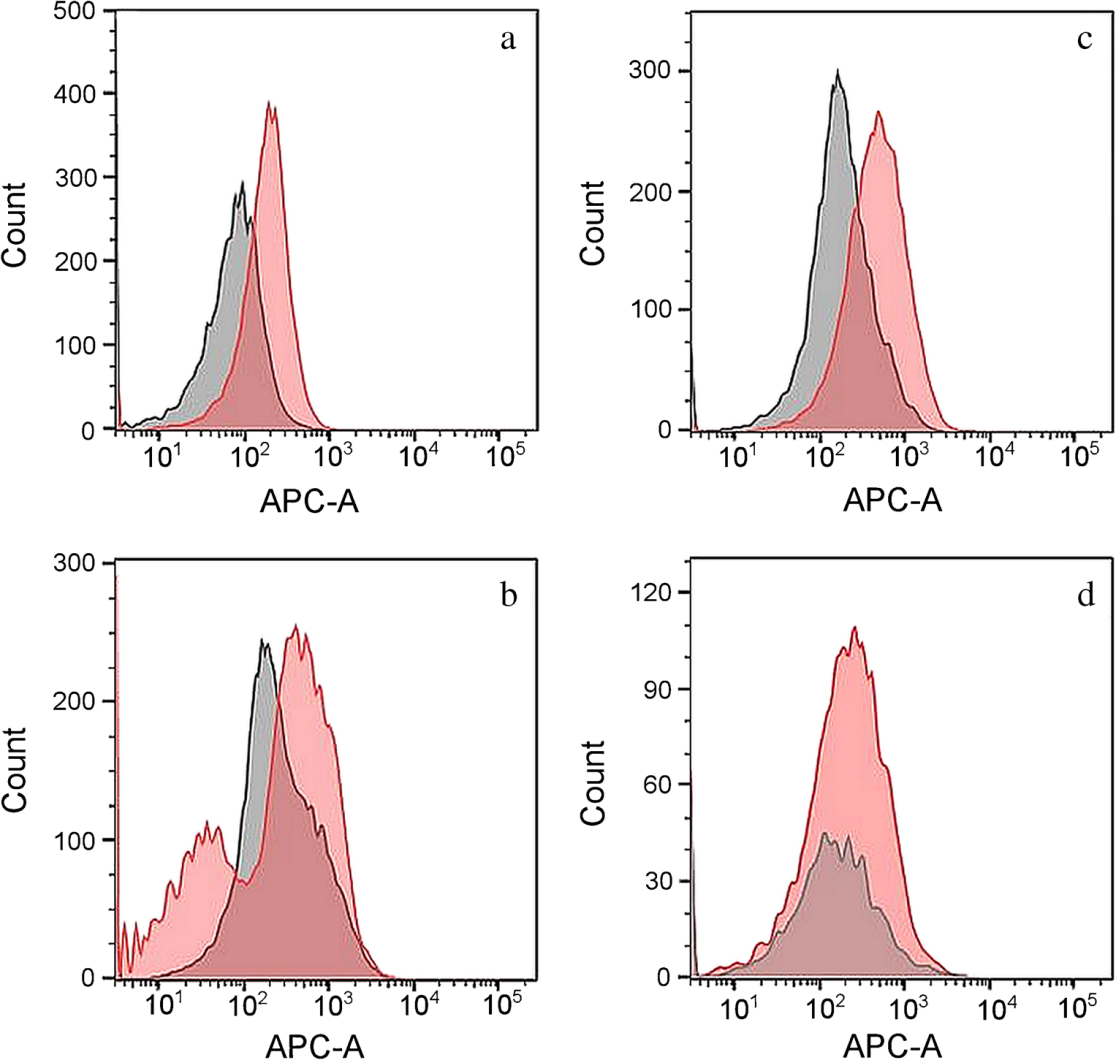
Overlaid APC channel histograms for cell populations generated from donors that wore a purple nitrile glove on one hand. The *red histogram* shows fluorescence data collected from cells derived from the hand that wore a purple nitrile glove, and the *gray histogram* shows fluorescence data collected from the control (bare) hand. Panels **a**–**c** represent three different contributors. Panels **c** and **d** are cell populations of the same contributor sampled on different days

Histograms of touch cell samples collected from donors who wore a purple nitrile glove on one hand also displayed slight shifts in mean fluorescence intensity compared to cell populations from the control (bare) hand, although considerable overlap from the two cell populations was observed (Fig. 3a–c). For samples collected from a single donor on different days, fluorescence distributions of test and control cell populations varied (Fig. 3c vs. Fig. 3d). The high degree of overlap observed in Fig. 3d appears to be attributable to the combined effects of lower intensity fluorescence of gloved hand (note the subset of cells fluorescing below 100 RFU, compared with Fig. 3c) and the relatively low cell yield from the ungloved hand. This further highlights the interplay between cell yield and fluorescence distributions underlying the ability to successfully sort (and type) such samples.

**Fig. 4:**
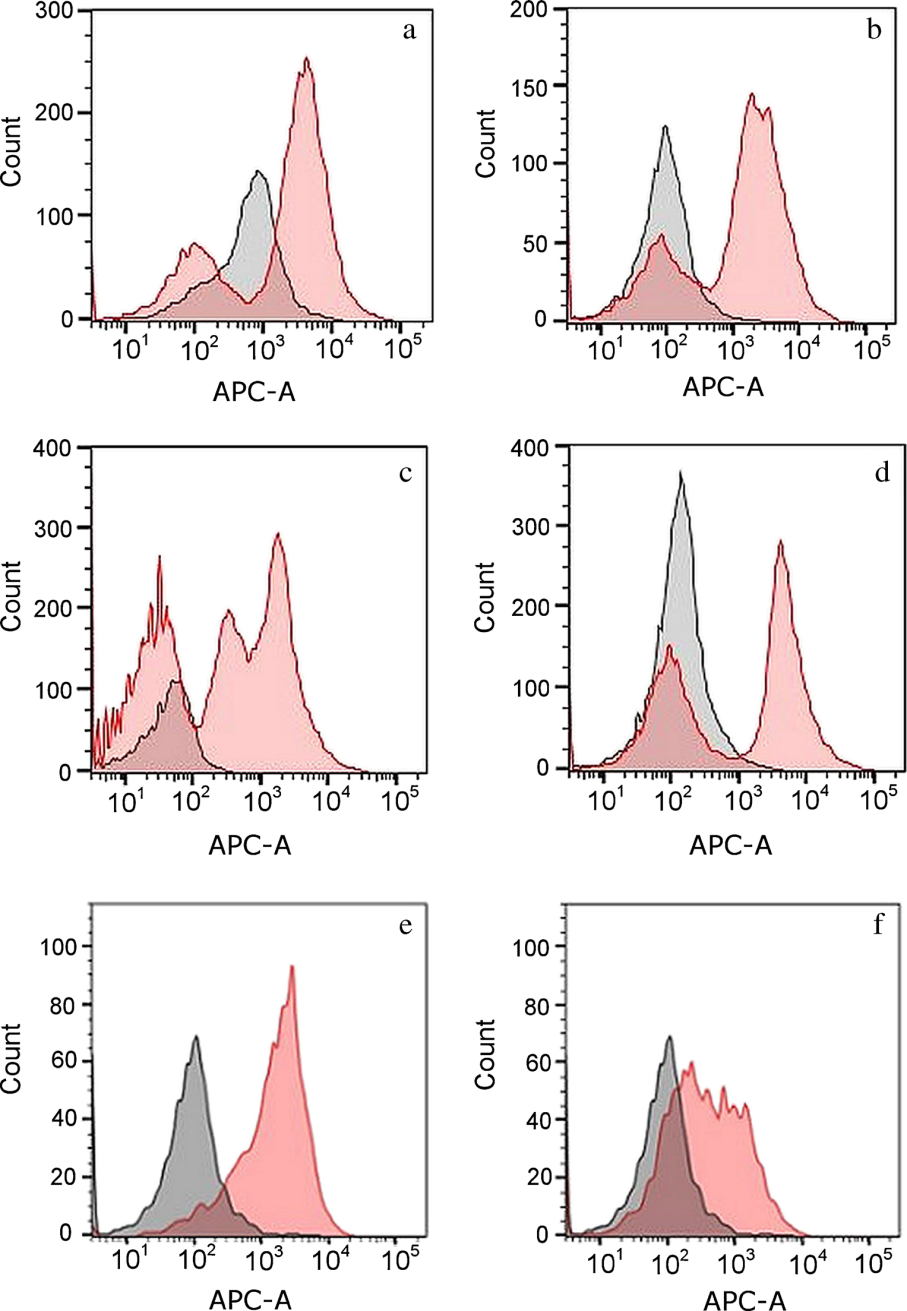
Overlaid APC channel histograms for samples generated from donors handling plant material (kale or collard green leaves) prior to cell deposition. Panels **a**–**d** show cell populations from the right and left hand of four different contributors that handled plant material with only one hand (*red histogram*) leaving the other hand as a control (*black histogram*). Panels **e** and **f** show overlaid APC channel histograms of contributor cell populations from one donor (same donor shown in panel **a**) before (**e**) and after (**f**) washing hands, again with the *red histogram* representing the test (kale-holding) hand and the *black histogram* representing a control sample collected on a different day

We observed much more distinct shifts in red autofluorescence intensity after donors handled plant material (kale or collard greens) relative to control cell populations derived from hands that had not handled plant material (Fig. 4a–d). A subset of cells from the plant-holding hand displayed lower levels of fluorescence, on par with control cell populations; this trend was observed in samples from four different donors, although the number of cells in the subset fluorescing at lower intensity varied across donors (e.g., compare Fig. 4a with Fig. 4c). This is likely attributable to the lack of precise control over how study participants handled plant material, which would be expected to result in a variable amount of transfer of plant material to the palms. Notably, one donor’s control touch sample showed a higher mean fluorescence compared not only to other donors’ control samples but also to the low-fluorescence subset of cells from that donor’s test (i.e., kale-handling) hand (Fig. 4a). One potential explanation is the control hand made contact with some unknown exogenous fluorescent material prior to this experiment, which persisted through initial hand washing. This underscores the difficulty of controlling for all conditions that could influence a touch sample (e.g., Fig. 3b) (control hand in nitrile glove experiment displays a secondary fluorescence “peak” around 1000 RFU suggesting a subset of palmar cells potentially associated with unknown exogenous fluorescent material). Another possibility is an endogenous influence, though the difference observed between the low-fluorescence cells from the two hands undercuts a biological basis, which would generally be expected to affect both hands similarly.

Introducing a hand washing step subsequent to handling plant material allowed us to examine the persistence of transferred fluorescence in touch samples. A distinct rightward shift in mean fluorescence comparable to that seen in cells derived from unwashed hands was observed to persist in touch samples collected after donors washed their hands with soap and water for 15–20 s (compare red histograms in Fig. 4e (pre-wash)) and Fig. 4f (post-wash)). However, a subset of post-wash cells were observed to fluoresce at the lower intensities characteristic of control (non-plant-handling) touch samples; this suggests that washing removed fluorescent substances from a portion of the cells in the sample.

**Fig. 5:**
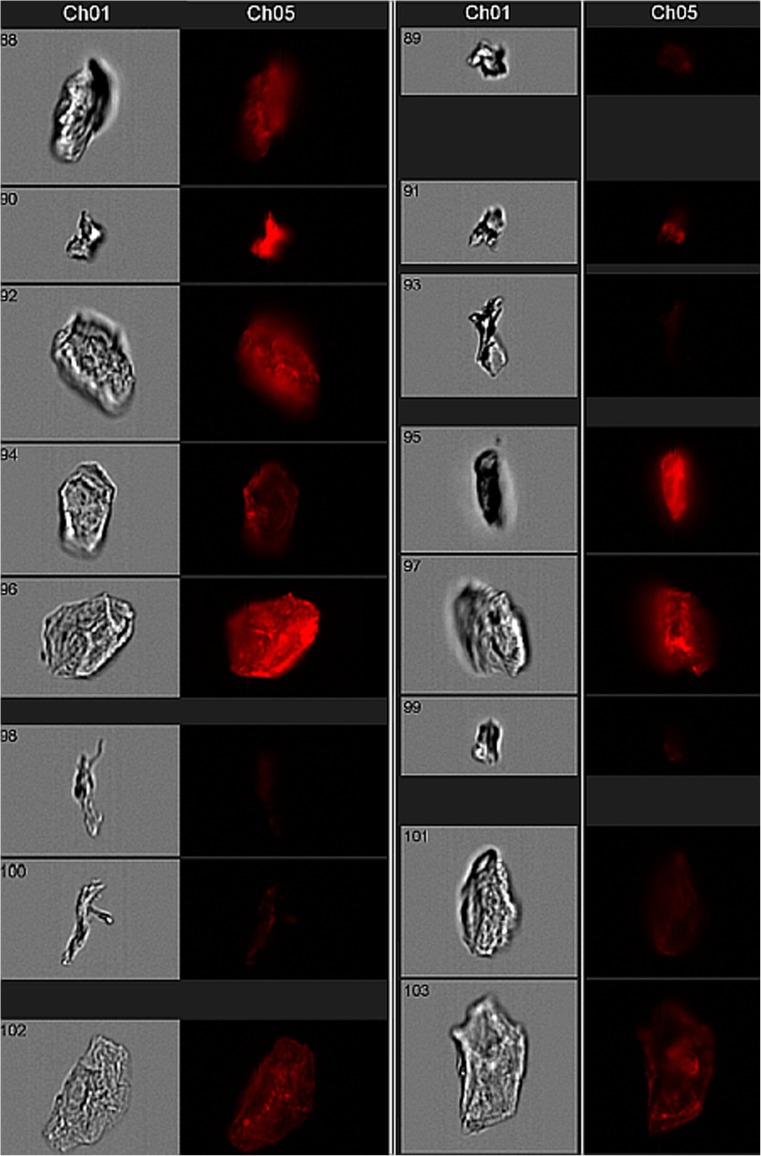
Amnis imaging of individual flow cytometry events from the fluorescent cell population shown in Fig. 4e (*red histogram*). Each event was visualized in two different microscopic settings: Brightfield (left image in *gray*) and APC channel fluorescence (right image in *black* background). The *X*-axis of each frame is 20 μm

Microscopic surveys of individual events derived from the cell population represented in the red histogram from Fig. 4e (donor I66) show that the fluorescence is associated with whole, intact cells consistent in size and morphology with keratinocytes (Fig. 5) and to a lesser extent with other material in the sample that may be rolled cells, cellular debris, or even non-cellular material. Similar fluorescence configurations (i.e., fluorescence primarily associated with apparent cell surfaces) were observed in microscopic surveys of donor E15’s cell samples from our initial touch studies, where there was no deliberate touching of particular materials, and the source of fluorescence is unknown (ESM Fig. S2).

Taken together, these results suggest that the intensity of red autofluorescence in contributor cell populations can be influenced by a person’s immediate prior contact with particular materials. Fluorescent compounds from handled materials may be transferred to and essentially “tag” cells of the palm and in turn may be transferred, in association with those cells, to touched objects. The known fluorescent properties of aromatic chlorophylls make these compounds a compelling candidate for the source of observed autofluorescence in experiments involving plant material. It should be noted that contributor cell populations in Fig. 4 also exhibited fluorescence at shorter wavelengths (e.g., 488 nm), consistent with chlorophyll compounds which exhibit autofluorescence across a wide range of wavelengths [23].

The compound responsible for the less pronounced increase in red autofluorescence intensity observed in experiments involving purple nitrile gloves is more ambiguous. Notably, we investigated this phenomenon further by having donors wear other gloves found in our laboratory, including blue nitrile and latex; associated touch samples did not exhibit increased red fluorescence. This may indicate that there is some substance unique to the purple nitrile gloves or that particular commercial brand that is the source of observed fluorescence. Laboratory gloves may be treated with a variety of additives, synthetic preservatives, or antimicrobials to facilitate removal, maintain shelf life, and/or ensure sterility [24] which may potentially impart fluorescence. Similarly, colored marker ink may contain a variety of components that contribute to fluorescence emissions within the visible spectrum including dyes, pigments, and fluorescers [25] which has been investigated within forensic contexts [26].

The day-to-day profile variability shown in Fig. 1 (where no particular materials were deliberately handled) suggests that there may be multiple factors or compounds contributing to the red autofluorescence signature. The frequency, duration, and type of contact a contributor has with various exogenous substances are likely to dictate the intensity and persistence of red autofluorescence (as well as autofluorescence at other wavelengths) in cells left by touch. We also cannot discount the possibility that differences in epidermal cell biology or palmar characteristics (e.g., sweat levels) between individuals may contribute to the persistence of exogenous materials on keratinocyte populations.

While the above experiments suggest that a donor’s contact with specific substances may influence the fluorescent properties of subsequently sloughed keratinocyte cell populations, this does not preclude the possibility that intracellular components may also contribute to this effect. As discussed previously [19], there are a number of endogenous molecules within the stratum corneum that can contribute to autofluorescence [27], including molecules such as porphyrins which have emission maxima similar to what was observed in this study [18, 28]. Understanding the factors, both intrinsic and extrinsic to the cell, which may cause shifts in autofluorescence in touch cell populations will be an important area of future research.

**Fig. 6:**
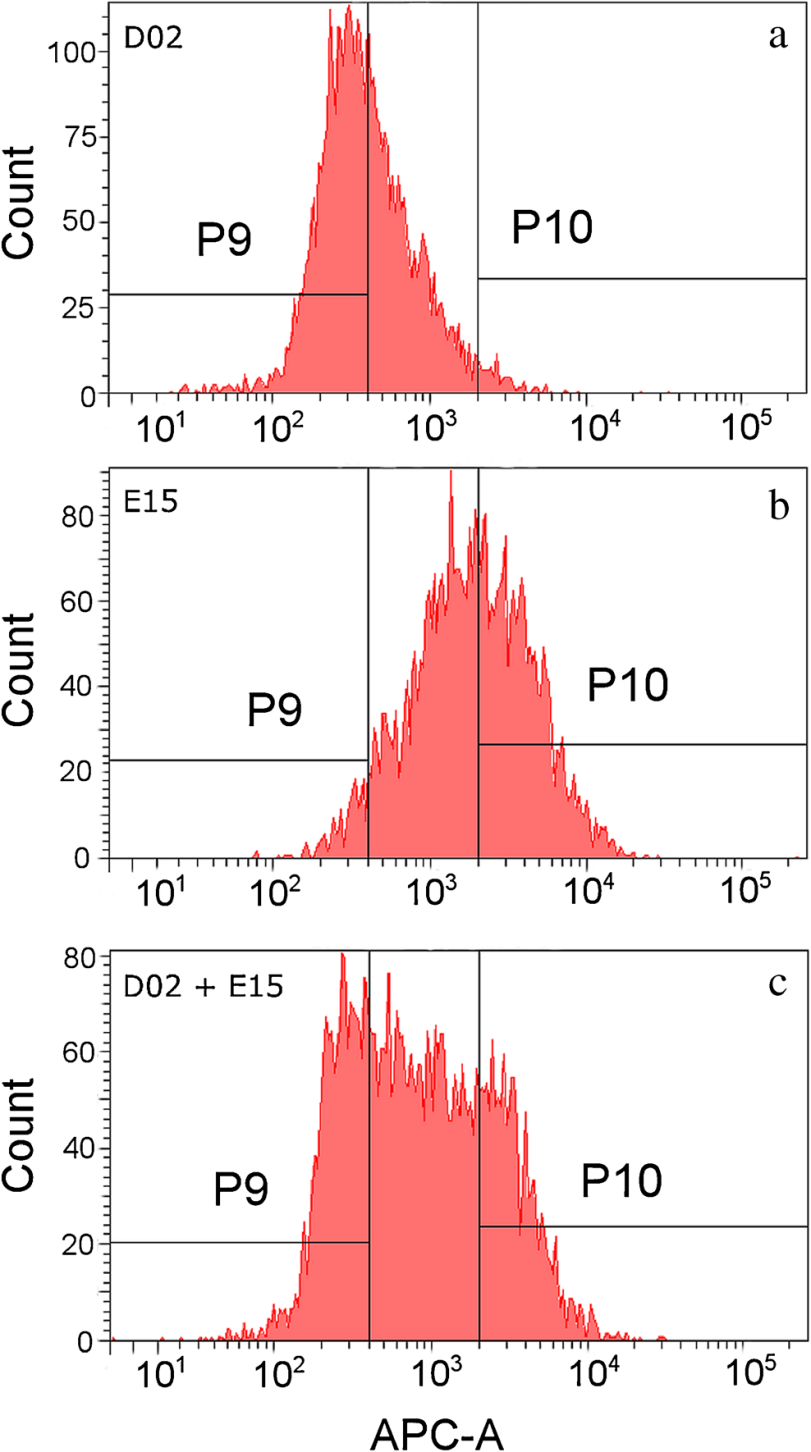
Sorting gates used for FACS based on APC channel intrinsic fluorescence. Histogram profiles for single source samples (panels **a**, **b**) were used to define two sorting gates, P9 and P10. These gates were positioned such that cell populations from D02 and E15 would be enriched relative to each other in the two cell fractions. Panel **c** shows the sorting gates plotted against the histogram profile of the two-person cell mixture prior to sorting

Ultimately, our observations regarding variations in red fluorescence in touch deposits suggest that there will be some touch cell mixtures that are more susceptible to being separated into individual components (or at least broken down into less complex cell mixtures) based upon this characteristic than others. Because flow cytometry is non-destructive, evidence samples could potentially be screened for favorable fluorescence distributions. A mixed cell sample that exhibits two or more peaks (e.g., Fig. 6c) on a fluorescence histogram may be a more promising candidate for cell separation than one that exhibits a unimodal fluorescence distribution (e.g., Fig. S3c, see ESM), as the latter suggests a high degree of overlap between contributor cell populations. Our preliminary results appear to bear out this proposition (compare Table 1 and ESM Table S1), but further research is required to develop a standardized set of screening criteria.

**Table 1:** STR profiles developed from sorted fractions of two-person mixture

Locus	Sort A^a^	Sort B^b^	Unsorted mixture	D02 reference^c^	E15 reference^c^
D3S1359	15	15, 17	15, 17	15	15, 17
D1S1656	12	–	12, 13, 16.3	12, 17.3	13, 16.3
D2S441	–	–	11.3, 14	14, 15	10, 14
D10S1248	–	–	–	15, 16	13, 14
D13S317	–	13	–	11, 14	9, 11
Penta E	–	–	–	10, 18	16, 21
D16S539	13	10	10, 11, 12, 13, 14	11, 13	10, 12
D18S51	13	–	13, 18, 20	13, 14	13, 18
D2S1338	24	25	17, 18, 20, 25	18, 20	19, 25
CSF1PO	–	–	10	11, 12	11, 13
Penta D	–	–	–	11, 15	11, 13
TH01	–	7	7, 9	9	7
vWA	–	16	17, 18	18, 19	16, 17
D21S11	–	–	–	28, 33.2	30, 31.2
D7S820	–	–	12	8, 12	12
D5S818	–	–	–	12, 13	11, 12
TPOX	–	–	8	8	8, 12
DYS391	–	–	–	–	11
D8S1179	12, 13	–	8, 12, 13, 14, 16	12, 13	14, 16
D12S391	19	–	18, 19, 20, 21	19, 21	18, 20
D19S433	14, 15	14	14, 15	14, 15	14, 16.2
FGA	–	–	23, 25, 27	24, 25	25, 27
D22S1045	–	–	16	15, 16	15

^a^Cell fraction that met the gating criteria based upon D02’s intrinsic fluorescence

^b^Cell fraction that met the gating criteria based upon E15’s intrinsic fluorescence

^c^Buccal reference samples

### Two-person mixture study

Even a touch mixture composed of readily distinguished cell populations may not necessarily separate cleanly or produce worthwhile STR data. Our group and others have reported on the characteristically low levels of intracellular genomic DNA recovered from cells deposited on touch surfaces [20, 29], which is expected given that keratinocyte differentiation involves programmed breakdown of nuclear DNA prior to cell shedding from the stratum corneum [11, 30]. This could pose a challenge for the application of cell-based separation techniques on touch samples, particularly those composed exclusively of skin cells.

With that in mind, we utilized FACS to sort a controlled touch mixture of two donors (D02 and E15) whose touch samples displayed distinct differences in red fluorescence in initial experiments (where neither donor deliberately handled known autofluorescent substances before depositing cells). We then attempted DNA analysis of the resultant low-fluorescence and high-fluorescence fractions using a standard forensic workflow. The fluorescence histogram of the cell mixture, overlaid with the sorting gates utilized to capture these fractions, is shown in Fig. 6. After sorting, cell populations were subjected to DNA analysis. “Sort A” and “sort B”—the cell fractions that met gating criteria derived from intrinsic fluorescence measurements of cells from donors D02 and E15 (respectively)—each produced a partial profile (Table 1). The high degree of dropout and possible drop-in alleles observed are consistent with the extremely low level of template DNA detected in each cell fraction (<50 pg). Notably, these low DNA levels were obtained from fractions in which more than 9000 “large cell” flow cytometry events were sorted from the mixture. This is consistent with our earlier studies showing that flow-derived cell yield for touch samples is not a good predictor of ultimate DNA yield [20].

All alleles detected in sort A were consistent with donor D02 with the exception of a single 24 allele at locus D2S1338, which did not originate from donor E15 and is likely a drop-in allele; none of E15’s obligate (i.e., unique) alleles were detected in the DNA profile developed from sort A. Likewise, all alleles detected in sort B were consistent with donor E15 with the exception of a single 13 allele at locus D13S317, which did not originate from donor D02 and is likely a drop-in allele; none of D02’s obligate alleles were detected in the DNA profile developed from sort B.

These preliminary efforts—resulting in a partial STR profile for each sorted touch fraction that is (with the exception of a single extraneous allele) consistent with the respective known contributor—indicate that separation of cell populations from the two known contributors on the basis of red autofluorescence was successful. However, the single stray allele in each sort suggests that a very low level of DNA from a third party may have ended up in these fractions. Given the low levels of target template, it is possible that these are examples of allelic drop-in during amplification; negative controls were clean but this does not exclude the possibility of this phenomenon. Interestingly, six extraneous alleles (i.e., not from D02 or E15) were detected in the unsorted mixture (Table 1). None of these alleles showed up in profiles developed from sort A or sort B. These could be instances of drop-in (11.3 at D2S441, 20 at D18S51, and 8 at D8S1179) and pronounced stutter (17 at D2S1338, 14 at D16S539, and 23 at FGA) resulting from low levels of DNA template in the touch mixture. It is also possible that these alleles are derived from extracellular DNA (which would not be expected to show up in sorted fractions) that was transferred to the palms of D02 or E15 before they deposited their touch samples, particularly in light of studies demonstrating the prevalence of extracellular DNA in touch samples [20, 29, 31].

In general, the high degree of allelic dropout observed in sorted cell fractions is not unexpected given the nature of the biological material being analyzed—primarily shed epidermal cells. However, future work should test methodological adjustments to improve DNA yield and/or maximize the use of the DNA that is present and thus produce more complete DNA profiles from sorted fractions. For example, we utilized a standard forensic DNA analysis protocol on sorted samples, which could be modified in various ways to increase efficiency (e.g., by reducing extract volume and/or concentrating post-quantitation). Moreover, these controlled touch mixtures were split into aliquots to be used for differing purposes during these exploratory studies (e.g., microscopic imaging, FACS, DNA analysis without sorting). If more (or all) of each touch sample was utilized for FACS, each fraction would likely contain more cells for downstream STR profiling.

Further, by designing the sorting gates in this study with an eye toward producing single source profiles, we sacrificed maximal cell recovery for purity of the sort. As can be seen from Fig. 6, gate P9 was designed to capture D02’s cells while excluding most of E15’s cells, and gate P10 was designed to capture E15’s cells while excluding most of D02’s. However, approximately half of each of D02 and E15’s cells went unsorted in the middle area between the two gates. Based on these results and the associated difficulties related to intracellular DNA yield from corneocytes, it may be necessary to shift the gating calculus. Instead of designing gates to produce single source profiles, one might strike a balance between cell recovery and production of simple mixtures with easily discernable major components (e.g., applying a gate that sorts all cells in the Fig. 6c mixture fluorescing less than 1000 RFU into sort A and those fluorescing at or greater than 1000 RFU into sort B).

One of the biggest drivers of cell loss in our methodology may be the retention of cellular material in the collection swabs following manually stirring and vortexing in water to elute the cells into solution. The challenge of maximizing DNA yield from collection swabs has been explored by a number of researchers in the forensic sciences, though many of the protocols are not applicable where, as here, cells need to remain intact during elution. Future work should continue to test different collection (e.g., scraping, tape-lift) and elution protocols to maximize cell recovery; optimized buffers [32] and the incorporation of enzymes such as cellulase to break down cotton and encourage the release of cells [33] may hold promise. At very least, this unsorted mixture data may be used to give context to STR profiles developed from sorted cell fractions; in some cases, the combination of sorted and unsorted DNA data may increase the overall probative value of a sample.

Finally, because a significant portion of the genetic material in many touch samples may be unavoidably extracellular, characterizing the chemical and physical relationship between cell-free DNA and the surface of intact epidermal cells may be an important area of future research. If extracellular DNA associates with epidermal cells, as it has been observed to do in other cell types [34], flow cytometry protocols could potentially be optimized to maintain surface-bound DNA through the cell sorting process. If it emerges that extracellular DNA is not bound to epidermal cells at the time of transfer, or is not bound in a way that can be maintained through the sorting process, this DNA source can be separately collected for typing (e.g., the workflow described in [20]).

## Conclusions

This investigative study marks a starting point for ongoing research into methods that facilitate the separation of touch samples into individual contributor cell populations for downstream DNA analysis. While additional research is needed before FACS can be imported as a front end technique in forensic DNA casework, our results indicate that there are features of fully differentiated keratinocytes—be they endogenous, exogenous, or both—that can be harnessed to distinguish cell populations from some individuals. A benefit of a feature such as red autofluorescence is that it can be measured without the need for antibody probes or other special reagents, allowing for touch samples to be easily pre-screened for this feature.

Maximizing both cell yield and DNA yield from sorted cell populations will likely continue to be a challenge. However, the recovery of even partial profiles from sorted cell solutions may have the potential to enhance the overall probative value of DNA evidence, particularly when analyzed in conjunction with complex mixture data derived from the same sample (e.g., considered alongside profiles generated from the extracellular fraction and/or cells retained in swabs). Sorted profiles, even if too incomplete to stand alone, may be able to buttress probabilistic claims about the mixture. Alternatively, instead of aiming for single source profiles, complex mixtures may be sorted into simpler mixtures (i.e., easily discernible major-minor). At very least, partial profiles have the potential to provide important investigatory leads, e.g., by supplying clues as to allelic pairings in an otherwise indistinguishable mixture and thereby narrowing the pool of potential contributors.

## Electronic supplementary material

ESM 1 (PDF 608 kb)
