# Supplementary material for: Analysis of cellular autofluorescence in touch samples by flow cytometry: implications for front end separation of trace mixture evidence
Source: Anal Bioanal Chem. 2017 May 18;409(17):4167–79. doi: 10.1007/s00216-017-0364-0 (PMC5486938; doi:10.1007/s00216-017-0364-0)
Supplement: Supplementary file 1 — (PDF 608 kb) [file 216_2017_364_MOESM1_ESM.pdf]

Analytical and Bioanalytical Chemistry

Electronic Supplementary Material

Analysis of cellular autofluorescence in touch samples by flow cytometry: implications for front end separation of trace mixture evidence

M. Katherine Philpott, Cristina E. Stanciu, Ye Jin Kwon, Eduardo Bustamante,
Susan Greenspoon, Christopher J. Ehrhardt

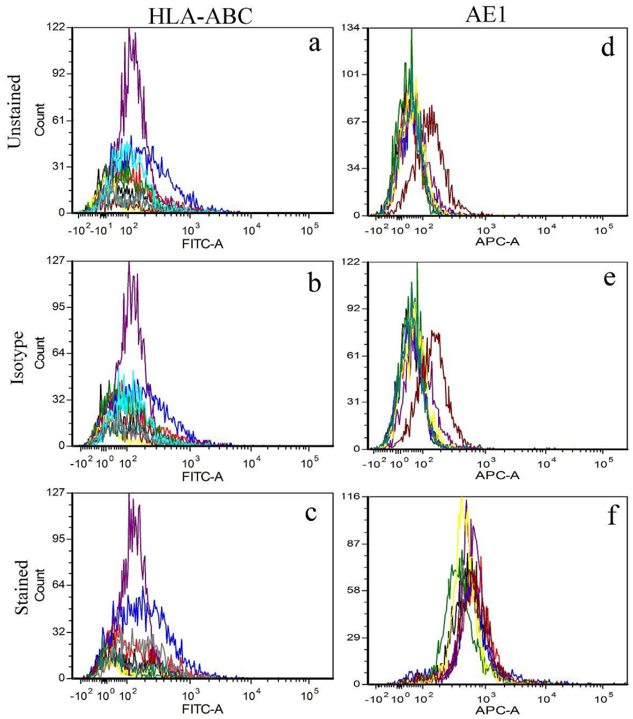

Fig. S1 Hybridization of touch samples with HLA and CK antibody probes. Few differences were observed between samples hybridized with pan-HLA probe and unstained samples/isotype controls, indicating that the touch samples failed to uptake the probe (panels a-c). In contrast, all touch samples exhibited uptake of AE1 cytokeratin antibody probe, with slight differences observed in binding efficiency across contributor cell populations (panels d-f)

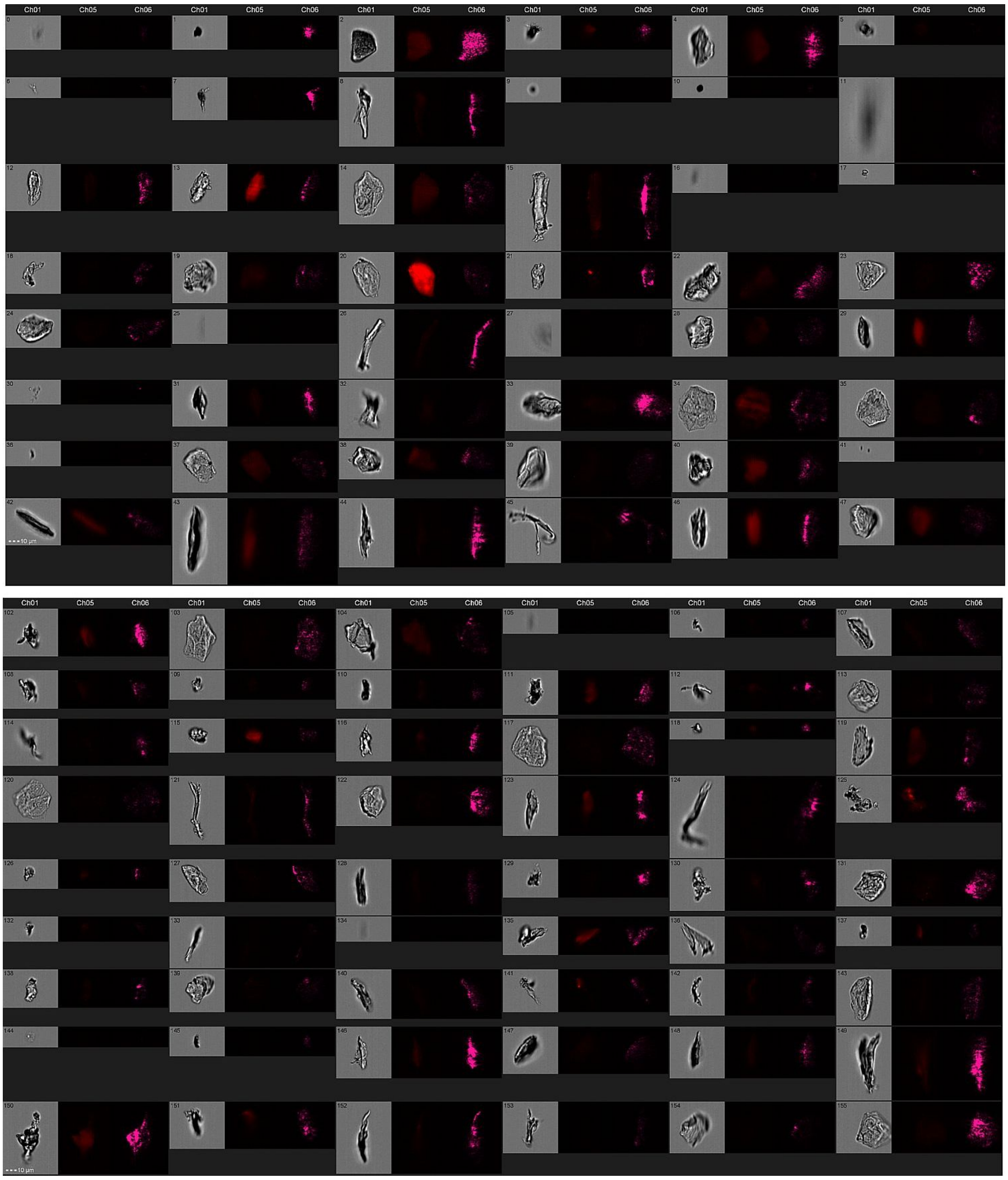

Fig. S2 Amnis imaging of individual flow cytometry events from the “large cell” fraction of touch samples from two different contributors, E15 (top) and D02 (bottom). Each event was visualized in three different microscopic settings: Brightfield (left image in gray), APC channel fluorescence (middle image shown in red), and side scatter (right image shown in purple)

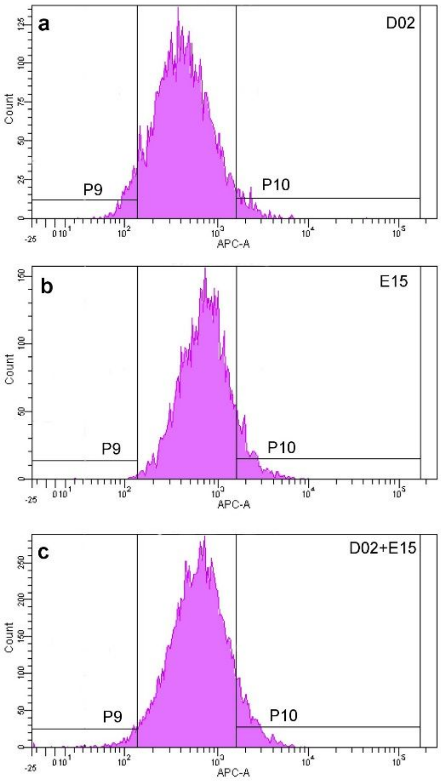

Fig. S3 APC-channel histograms of touch samples from donor D02 (a), donor E15 (b), and mixture of touch samples from D02 and E15 (c). This mixture was created on a day when the fluorescence histograms of touch samples from D02 and E15 displayed a high degree of overlap. Histograms (a) and (b) were used to define two sorting gates, P9 and P10, designed to enrich cells from D02 and E15 (respectively). Panel c shows the more unimodal fluorescence histogram profile of the mixed sample (compared to histogram distribution observed in Figure 8c), overlayed with the pre-defined gates. Because most of the cells from D02 and E15 possess overlapping fluorescence characteristics, these gates capture a small fraction of the total sample (much smaller than the gates displayed in Fig. 8)

Table S1 Powerplex fusion profiles of FACS-separated fractions from D02-E15 cell mixture (shown in Fig. S3), alongside reference samples for D02 and E15

LOCUS	Sort A	Sort B	D02 Ref	E15 Ref
**D3S1359**	-	15	15	15, 17
**D1S1656**	-	-	12, 17.3	13, 16.3
**D2S441**	-	10, 14	14, 15	10, 14
**D10S1248**	-	13	15, 16	13, 14
**D13S317**	-	-	11, 14	9, 11
**Penta E**	-	-	10, 18	16, 21
**D16S539**	10	10	11, 13	10, 12
**D18S51**	13	-	13, 14	13, 18
**D2S1338**	-	-	18, 20	19, 25
**CSF1PO**	-	-	11, 12	11, 13
**Penta D**	-	-	11, 15	11, 13
**TH01**	-	7	9	7
**vWA**	-	16	18, 19	16, 17
**D21S11**	-	-	28, 33.2	30, 31.2
**D7S820**	-	-	8, 12	12
**D5S818**	-	-	12, 13	11, 12
**TPOX**	-	-	8	8, 12
**DYS391**	-	-	-	11
**D8S1179**	13	16	12, 13	14, 16
**D12S391**	-	-	19, 21	18, 20
**D19S433**	-	-	14, 15	14, 16.2
**FGA**	-	-	24, 25	25, 27
**D22S1045**	-	-	15, 16	15

In contrast to the samples depicted in Fig. 6, these samples were extracted in a different laboratory via a traditional organic method, and interpreted using that laboratory's 50 RFU peak amplitude threshold. "Sort A" is the cell fraction that met the P9 gating criteria and "Sort B" is

the cell fraction that met the P10 gating criteria. A high level of dropout is observed in each fraction, which is not surprising given the small percentage of the total sample captured by the gates. Note that Sort B was much more successful at capturing E15's DNA (8 alleles detected, all consistent with E15) than Sort A was at capturing D02's DNA (3 alleles detected, 2 consistent with D02). Given the number of cells from D02 in Sort A should have been roughly comparable to the number of cells from E15 in Sort B, the disparate results may be a consequence of different degrees of degradation (and hence intracellular DNA content) of the donors' cells.
